# Evolution of *Homo* in the Middle and Late Pleistocene

**DOI:** 10.1016/j.jhevol.2022.103279

**Published:** 2022-12

**Authors:** Katerina Harvati, Hugo Reyes-Centeno

**Affiliations:** a Paleoanthropology, Institute for Archaeological Sciences and Senckenberg Centre for Human Evolution and Palaeoenvironment, Eberhard Karls University of Tübingen, Rümelinstrasse 19-23, Tübingen 72070, Germany; b DFG Centre for Advanced Studies ‘Words, Bones, Genes, Tools: Tracking Linguistic, Cultural and Biological Trajectories of the Human Past’, Rümelinstrasse 19-23, Tübingen 72070, Germany; c Department of Anthropology, University of Kentucky, 211 Lafferty Hall, Lexington, KY 40506, USA; d William S. Webb Museum of Anthropology, University of Kentucky, 1020 Export St, Lexington, KY 40504, USA

**Keywords:** *Homo heidelbergensis*, Neanderthals, Modern human origins, Speciation, Hybridization

## Abstract

The Middle and Late Pleistocene is arguably the most interesting period in human evolution. This broad period witnessed the evolution of our own lineage, as well as that of our sister taxon, the Neanderthals, and related Denisovans. It is exceptionally rich in both fossil and archaeological remains, and uniquely benefits from insights gained through molecular approaches, such as paleogenetics and paleoproteomics, that are currently not widely applicable in earlier contexts. This wealth of information paints a highly complex picture, often described as ‘the Muddle in the Middle,’ defying the common adage that ‘more evidence is needed’ to resolve it. Here we review competing phylogenetic scenarios and the historical and theoretical developments that shaped our approaches to the fossil record, as well as some of the many remaining open questions associated with this period. We propose that advancing our understanding of this critical time requires more than the addition of data and will necessitate a major shift in our conceptual and theoretical framework.

## 1 Introduction

The Middle to Late Pleistocene is highly relevant for the study of human origins. This is the period when the ancestral lineage of living humans developed, thought to have first emerged as early as ca. 300 ka in Africa from both fossil (Hublin et al., 2017) and genetic (Schlebusch et al., 2017; Lipson et al., 2020) evidence. It also witnessed the evolution of the Neanderthals, an iconic Pleistocene Eurasian species. Their trajectory is relatively well documented through a comparatively abundant record comprising partial fossil skeletons seemingly preserved through intentional burial, as well as a wealth of associated archaeological remains and, more recently, paleogenetic information and even complete genomes. The convergence of the Neanderthal and modern human lineages and its aftermath, starting at ca. 60 ka with the major dispersal of modern humans out of Africa and concluding with the extinction of Neanderthals ca. 39 ka, is also relatively well understood—even if the primary driving forces behind it (e.g., demographic, environmental, cultural, cognitive) are still intensely debated.

Beyond these areas of broad agreement lie more murky domains. Most importantly, it is unclear how many taxa or lineages are represented by the observed variation, how they were related to each other, and how the intensifying climatic oscillations of this period affected their diversification. While African *Homo erectus*, sometimes recognized as a different taxon, *Homo ergaster*, is commonly considered to have given rise to the precursors of both the *Homo sapiens* and *Homo neanderthalensis* lineages (e.g., Hublin, 2009; Bergström et al., 2021), Asian *H. erectus* is thought to have persisted until the late Pleistocene (Rizal et al., 2020), possibly giving rise to the small-bodied insular taxa identified in Flores, Indonesia (Brown et al., 2004), and, more recently, in Luzon, the Philippines (Détroit et al., 2019). The recently discovered small-brained *Homo naledi* in southern Africa, a possible descendant of African *H. erectus*, was revealed to date surprisingly late at ca. 300 ka (Berger et al., 2015; Robbins et al., 2021), greatly expanding the range of human variation that was contemporaneous, and possibly sympatric, with the earliest representatives of the *H. sapiens* lineage. The Middle to Late Pleistocene fossil record of Asia has long been recognized as complex, and the relationships of these fossils to either the *H. neanderthalensis* or the *H. sapiens* lineages is under intense discussion. The identification of a genetic lineage closely related to Neanderthals, dubbed the ‘Denisovans’ (Krause et al., 2010; Reich et al., 2010), has only recently been linked to fossil remains preserving more complete morphology (Xiahe, Tibetan plateau of China: Chen et al., 2019). Without informative anatomy preserved, it has not been possible to assign these mysterious hominins to taxon (but see Derevianko, 2011; Anagnostou et al., 2015; Zubova et al., 2017; Agadjanian and Shunkov, 2018). However, several candidates may represent this genetic form, including the recently described *Homo longi* (Ji et al., 2021; Ni et al., 2021) and other recent fossil finds from China (e.g., Xuchang: Li et al., 2017; Hualongdong: Wu et al., 2019, 2021), in addition to well-studied specimens, such as e.g., the Narmada cranium, India (Sonakia, 1985, 2007; Sankhyan, 2007; Athreya, 2011; Kennedy, 2014), or the Maba or Jinniushan fossils from China (Woo and Peng, 1959; Wu, 1988; 2017; Wu and Bruner 2016).

In this tangle of proposed hominin species, no taxon is more contentious than *Homo heidelbergensis*. Much ink has been spilled trying to evaluate the integrity, or even justify the existence, of this taxon (e.g., Harvati, 2007; Mounier et al., 2009; Stringer, 2012; Athreya and Hopkins, 2021). Its diagnosis is complicated by the fact that its type specimen, the Mauer fossil from Germany, is a mandible (Schoetensack, 1908); and because it is not defined by uniquely derived features of its own, but rather by cranial morphology perceived to be intermediate between earlier (*H. erectus* s.l.) and later *Homo*. Much of this anatomy is related to the common trend of encephalization observed in both the Neanderthal and modern human lineages. *H. heidelbergensis* is commonly referred to as sensu lato or sensu stricto, to denote the different hypodigms attributed to it by different researchers. Sensu stricto refers only to European forms which have been linked to the morphology of the Mauer mandible; while sensu lato includes morphologically similar individuals from Africa and Asia. The latter has often been proposed as the common ancestor of the *H. sapiens* and *H. neanderthalensis* lineages (e.g., Stringer, 1983; Rightmire, 1998), although this view has been repeatedly questioned (e.g., Bermúdez de Castro et al., 1997; Arsuaga et al., 2014; Bermúdez de Castro et al., 2017). Still, the European *H. heidelbergensis* fossils are commonly considered ancestral to Neanderthals, showing ‘incipient’ Neanderthal diagnostic features, while the African counterparts have been proposed as ancestors of *H. sapiens*. In light of this, researchers have proposed sinking *H. heidelbergensis* s.s. into *H. neanderthalensis*, or preserving it only as a chronospecies of that lineage, and attributing the African fossil material to a different taxon, *Homo rhodesiensis*, considered ancestral to *H. sapiens* (e.g., Hublin, 2009). The recently obtained geological age of ca. 300 ka for the type specimen of *H. rhodesiensis*, the Broken Hill (Kabwe) cranium from Zambia (Grün et al., 2020), as well as the reinterpretation of modern human-like facial morphology as ancestral, rather than derived for our species (e.g., Lacruz et al., 2019), have led some to question this taxon's ancestral status (Grün et al., 2020; Bergström et al., 2021). Reshuffling the hypodigm of a proposed (mostly) African Middle Pleistocene species suggested to be ancestral to *H. sapiens* and termed *Homo bodoensis*, as recently proposed by Roksandic et al. (2022), circumvents unfortunate colonial associations of the nomen *H. rhodesiensis*, but does not clarify these thorny taxonomic and phylogenetic questions (see also Delson and Stringer, 2022; Sarmiento and Pickford, 2022). This proposal does not address the question of polarity of the modern human facial morphology (derived vs. ancestral) and begs the question of why such a taxon needs a species designation, when it could be considered part of the *H. sapiens* lineage. Conversely, if modern human facial morphology is ancestral and the Middle Pleistocene African *H. heidelbergensis* s.l. (aka *H. rhodesiensis/H. bodoensis*) are too derived to be ancestral to *H. sapiens*, we are left with the irksome question of who in the known fossil record can actually be considered the Middle Pleistocene precursor to *H. sapiens*.

Here we will outline the historical developments and theoretical breakthroughs that have shaped our understanding of this period in the past and propose that resolving the ‘Muddle in the Middle’ will not be accomplished only through the simple addition of new data. Rather, we propose that, as has been the case historically, any advances in this endeavor will require a major shift in our conceptual and theoretical framework.

## 2 Historical and theoretical considerations

At the heart of discussions over the ‘Muddle in the Middle’ are decisions about the apportionment of the observed variation and the diagnosis of species. This problem is a central one in biology, and one that is particularly intractable when dealing with the fossil record. When it comes to human evolution, considerable confusion exists in the literature, where explicit statements about the species concepts used to apportion variation in the fossil record are seldom made. Early workers, operating in the context of little or no known human fossil record, commonly emphasized morphological differences of individual fossils from modern humans (e.g., Gorjanović-Kramberger, 1906; Boule, 1911, Boule, 1912a, Boule, 1912b, Boule, 1912c, Boule, 1913; Peyrony, 1934) and often named taxa as specimens were discovered in widely disparate geographic and temporal settings (see, e.g., Tattersall, 2018). During this period several species were named, with varying standards of nomenclature applied, only to be abandoned later. This arbitrary and often typological approach to taxonomy was at first dictated by historical constraints related to the time and order of discovery, but continued well into the first half of the 20th century and, to a lesser extent, into more recent times (see, e.g., Szalay and Delson, 1979; Reed et al., 2022; Table 1).

**Table 1:** Species attributed to Middle to Late Pleistocene *Homo*.^a^^,^^b^

Species	Holotype	Holotype element(s)	Locality	Reference
*Homoaltaiensis*	(genomes of) Denisova 3, 4	(genomes of) phalanx, molar	Denisova Cave, Russia	Derevianko, 2011
*Homoantecessor∗*	ATD6-5	partial right mandible and teeth	Gran Dolina, Atapuerca, Spain	Bermúdez de Castro et al., 1997
*Homobodoensis∗*	Bodo 1	partial cranium	Bodo D'ar, Middle Awash, Ethiopia	Roksandic et al., 2022
*Homocepranensis∗*	Ceprano 1	calvaria	Campogrande, Ceprano, Italy	Mallegni et al., 2003
*Homofloresiensis∗*	LB1	partial skeleton	Liang Bua, Flores, Indonesia	Brown et al., 2004
*Homoheidelbergensis∗*	Mauer	mandible	Mauer, Germany	Schoetensack, 1908
*Homohelmei*	Florisbad	partial cranium	Florisbad, South Africa	Dreyer, 1935
*Homolongi∗*	HBSM2018-000018(A)	cranium	Upper Huangshan Formation, Harbin, China	Ji et al., 2021
*Homoluzonensis∗*	CCH6	postcanine maxillary teeth	Callao Cave, Philippines	Détroit et al., 2019
*Homomauritanicus*	Ternifine 1-2	partial mandibles	Ternifine (Tighenif), Algeria	Arambourg, 1954
*Homonaledi∗*	DH1	partial skeleton	Rising Star, South Africa	Berger et al., 2015
*Homonarmadensis*	U2-Hathnora	clavicles	Hathnora, India	Sankhyan, 2020
*Homoneanderthalensis∗*	Feldhofer 1	partial skeleton	Feldhofer, Germany	King, 1864
*Homonjarasensis*	Eyasi 1	partial cranium	Westbucht, Tanzania	Weinert et al., 1939
*Homorhodesiensis∗*	Broken Hill 1	cranium	Kabwe, Zambia	Woodward, 1921
*Homosaldanensis*	Saldanha	partial skull	Saldanha, South Africa	Drennan, 1955
*Homosapiens∗*	N/A	N/A	worldwide	Linnaeus, 1758
*Homosteinheimensis*	Steinheim	partial cranium	Steinheim, Germany	Berckhemer, 1936
*Homotsaichangensis∗*	Penghu 1	partial mandible	Penghu Channel, Taiwan	McMenamin, 2015

a This table shows key Middle-Late Pleistocene *Homo* fossils and species (in alphabetical order) that have been attributed to them, formally or informally. For a comprehensive list of nomina, see also, e.g., Szalay and Delson (1979) and Reed et al. (2022).

b An asterisk indicates that species is inscribed in ZooBank, the Official Register of the International Commission on Zoological Nomenclature.

The mid-20th century saw the development of the modern synthesis and the biological species concept (BSC; Mayr, 1942). The BSC is perhaps the most widely cited species definition in biology and postulates reproductive isolation as delimiting species boundaries. However, this process-based definition is difficult to operationalize, particularly in the fossil record, where reproductive behavior can no longer be observed. These theoretical developments had important repercussions for paleoanthropology (Tattersall, 2000; Foley, 2001; White, 2009; Wuketits, 2015). The influential review and ‘parsing’ of the human fossil record by Mayr, an evolutionary biologist and ornithologist by training (1950; see also Dobzhansky, 1944), led to the establishment and broad acceptance of low taxonomic diversity as the null model of human evolution. This assessment aimed to correct the arbitrary taxonomic practices common until that time in paleoanthropology and brought a much-needed emphasis on biological and evolutionary processes in the interpretation of the human fossil record (e.g., Howell, 1957). However, it also led to potential underestimation of taxonomic diversity in the genus *Homo* (e.g., Foley, 1991; Tattersall, 2000, 2018).

In the decades that followed, a consolidation of proposed taxa in the Middle to Late Pleistocene record was put forth. Specimens as far afield as China (Maba), Africa (Broken Hill, Omo 2, Jebel Irhoud) and Indonesia (Solo), were often attributed to a ‘Neanderthal stage’ in human evolution (e.g., Hrdlicka, 1927; Weidenreich, 1943; Brace, 1964, 1967; Brose and Wolpoff, 1971; but see Howell, 1957). In this view, such specimens were not considered taxonomically distinct, but instead broadly ancestral to modern humans and sometimes explicitly assigned to *H. sapiens* at a grade, subspecies, or even racial level (Brose and Wolpoff, 1971; Rightmire, 1976; Stringer et al., 1979; Wolpoff et al., 1997), as were also earlier parts of the hominin record (Wolpoff et al., 1994). This view often emphasized geographic and sexual, rather than phyletic, variation (Wolpoff, 1980). It contrasted sharply with the earlier ‘pre-*sapiens*’ hypothesis (Keith, 1915), which proposed distinct modern human and Neanderthal lineages of great antiquity in Europe; and was tightly intertwined with the multiregional model for modern human origins, which postulated a deep ancestry of *H. sapiens*, with ancient regional branches directly ancestral to modern human populations in the same geographic areas, and with continuous gene flow across continents maintaining species cohesion (e.g., Wolpoff et al., 1994).

In the late 1970s and gaining force in the 1980s and 1990s, the morphological diversity of the Middle to Late Pleistocene human fossil record came into focus once more and taxonomic and phylogenetic questions resurfaced. New fossil discoveries added to the record, and improved dating methods refined its temporal dimension, while the application of approaches rooted in cladistics (e.g., Delson et al., 1977, an influential article published in an early Journal of Human Evolution issue) and the phylogenetic species concept (PSC; Eldredge and Cracraft, 1980; Cracraft, 1983) gained traction (Stringer and Andrews, 1988; Kimbel and Rak, 1993). These perspectives helped clarify part of the Middle to Late Pleistocene human fossil record—even if the validity of applying cladistic methodology at the species level has been questioned (e.g., Szalay, 1993, 2001; White, 2014).

The phylogenetic species concept defines species as the smallest diagnosable unit of organisms that share an ancestral-descendant pattern. It is compatible with cladistic methodology but does not require phylogenetic analyses before it is applied. This concept, however, can underestimate within-species variation. In contrast, species definitions based on the possession of autapomorphic features of monophyletic groups require cladistic analysis before they can be applied (Nixon and Wheeler, 1990), thus resulting in a circular approach. Despite this shortcoming, this approach, even if not always explicit, has been greatly influential in paleoanthropology, as, for example, in the diagnosis of Neanderthals based on a suite of derived features (for a recent comprehensive review of proposed Neanderthal derived traits, see Harvati, 2015), starting with the breakthrough work of Santa Luca (1978), another seminal work from the early issues of the Journal of Human Evolution, which marked the departure from previous views of a ‘Neanderthal stage’ in human evolution (see also Hublin, 1978). However, defining autapomorphic features for Middle to Late Pleistocene human taxa is not always straightforward, as they often exhibit features with overlapping ranges of morphological variation between groups. Differences in the frequencies of particular character states, or combinations of features, rather than a strict criterion of presence or absence of autapomorphies, are therefore often accepted as defining characteristics for diagnosis (see, e.g., Stringer and Trinkaus, 1981; Day and Stringer, 1991; Bailey, 2006; Martinón-Torres et al., 2012).

The additional recognition that variation both below and above the species level should be considered when defining species boundaries in the fossil record (Tattersall, 1986) led to research aiming to place the Neanderthal-modern human morphological differences in the framework of primate interspecific and intraspecific diversity (Harvati, 2003; Harvati et al., 2004). This work highlighted the unusually high degree of cranial morphological differentiation between Neanderthals and modern humans, supporting a species-level distinction on phenetic grounds. Such a relatively high level of differentiation, combined with the extensive list of recognized autapomorphic features, was interpreted at the time as indicating reproductive isolation and as consistent with the replacement model for modern human origins. Concurrently, advances in DNA sequencing allowed for the analysis of mitochondrial DNA from the Neanderthal type specimen, Feldhofer 1, showing Neanderthals deeply divergent from living human populations and exceeding mtDNA differentiation compared to chimpanzee subspecies (Krings et al., 1997, 1999). These early paleogenetic studies on Neanderthals (see also, e.g., Ovchinnikov et al., 2000) were also interpreted to support a replacement model for modern human origins, nonetheless cautioning on the need for further sampling across the genomes of more Middle to Late Pleistocene fossils.

## 3 Where are we now?

The early 21st century opened yet another chapter in the study of the Middle to Late Pleistocene human fossil record, marked by the advent of paleogenomics and the Neanderthal genome project, but also by multiple unexpected sensational discoveries from the fossil record. Paleogenetic approaches not only identified repeated, if relatively rare, gene flow from Neanderthals into the modern human gene pool (e.g., Green et al., 2010; Fu et al., 2016; Villanea and Schraiber, 2019; Hajdinjak et al., 2021), but also recognized a previously unknown genetic lineage related to Neanderthals, dubbed ‘Denisovans’, in Asia (Krause et al., 2010; Reich et al., 2010; Meyer et al., 2012). Increasingly, episodes of admixture between the Denisovan, Neanderthal, modern human, and enigmatic ‘super archaic’ lineages have been proposed (Posth et al., 2017; Slon et al., 2018; Durvasula and Sankararaman, 2020; Prüfer et al., 2021), once more raising the question of alpha taxonomy in the context of interfertility. Although precisely inferring the relative magnitude of these processes over time remains difficult in the absence of sufficient ancient genomes and reliable demographic information, the low amount of Neanderthal and Denisovan DNA in modern human groups suggests that purifying selection acted strongly in the first generations after hybridization (Petr et al., 2019). Taken at face value, this pattern is suggestive of some level of genetic incompatibility (e.g., Sankararaman et al., 2014), leading some to propose possible reduced hybrid fitness (e.g., Currat and Excoffier, 2011), although the transfer of adaptive alleles through this process has also been documented (e.g., Huerta-Sánchez et al., 2014; reviewed in Racimo et al., 2015).

At the same time, new discoveries have kept upending received wisdom, demonstrating the fragmented and incomplete nature of our knowledge at every turn. Among the most prominent of these was the discovery of late surviving small-bodied and small-brained hominins in Southeast Asia (Brown et al., 2004; Détroit et al., 2019), demonstrating that hominins can be subject to evolutionary processes, such as island dwarfing, from which we formerly considered them exempt; the identification of apparent early forays of modern human ancestors into Europe (Harvati et al., 2019; Slimak et al., 2022), previously considered exclusive territory of Neanderthals before ca. 46 ka; and perhaps most astonishingly, the discovery of a small-brained hominin, named *H. naledi*, dating to as late as 300 ka in one of the best researched areas of the world, the Cradle of Humankind, South Africa (Berger et al., 2015; Dirks et al., 2017)—a species which would have existed contemporaneously, and perhaps alongside, early members of the *H. sapiens* lineage.

These developments highlight the diversity, co-existence, and likely sympatry of several hominin lineages in the Middle to Late Pleistocene, while at the same time underlining multiple instances of genetic exchange among at least some of them, toppling the assumption that (complete) reproductive isolation is a prerequisite for evolutionary distinctiveness, and challenging previous models of modern human origins based on either regional continuity or complete replacement (e.g., Holliday, 2003; Holliday et al., 2014; Smith et al., 2017). They have also prompted a re-examination of the role of hybridization in human evolution, a process that occurs at greater rates than previously appreciated even among commonly accepted extant mammalian species (e.g., Mallet, 2001; Holliday, 2003; Ackermann et al., 2019; Harvati and Ackermann, 2022). Recent work has found, for example, that more than 10% of all primate species engage in cross-species hybridization (Zinner et al., 2011), an observation that is difficult to reconcile with the provisos of the BSC. Furthermore, a survey of 146 hybrid crosses among eutherian mammals found that reproductive isolation tends to correlate with time of divergence, with interfertility among lineages dropping after 2 Ma of separate evolution (Holliday et al., 2014)—a much greater timeframe than current estimates of 500–700 ka for the divergence of modern humans from the Neanderthal and Denisovan lineages, or from other archaic lineages ca. 1 Ma (Bergström et al., 2021). Indeed, speciation itself is increasingly recognized as a process of continuous differentiation rather than an event, with morphological distinctiveness not necessarily coinciding with (complete) reproductive isolation, and with natural selection, rather than geographic isolation, as a potential driving factor (Mallet, 2001; Wu, 2001; Yoder, 2014; van Holstein and Foley, 2022). Such an understanding of speciation, emphasizing selection in addition to, or instead of, geographic isolation and decoupled from barriers to reproduction, might be the key to disentangling the Middle to Late Pleistocene human fossil record going forward.

Given the relatively recent diversification and interfertility of the Middle to Late Pleistocene human lineages, combined with their morphological distinctiveness maintained over time (at least in the case of *H. neanderthalensis* and *H. sapiens*), the concepts of ‘allotaxa’ (morphologically distinct but reproductively compatible taxa; Jolly, 2001), ‘syngamea’ (stable, cohesive species which maintain their identity across significant amounts of time even though they hybridize; Lotsy, 1925; Holliday, 2003) or ‘evolutionary lineages’ sensu Stringer (2016; distinct evolutionary units maintaining their identity through time despite low levels of introgression), rather than the BSC or the PSC, may be the most appropriate concepts to grapple with the diversity of the Middle to Late Pleistocene hominins. The application of these concepts eliminates the conflict between the criteria of interfertility vs. morphological/genetic differences and therefore also the debate, for example, of the species status of Neanderthals, who can be recognized as a distinct allotaxon or lineage. Such concepts, linked with a new understanding of speciation, would recognize the evolutionary significance of phenotypic distinctiveness maintained through time, established through a variety of evolutionary processes not necessarily requiring allopatry, including population structure, genetic drift, and natural selection; while accepting a degree of genetic permeability and lateral gene transfer as a potentially important element of lineage evolution (see, e.g., the adaptive nature of high-altitude relevant alleles contributed to modern humans by Denisovans; Huerta-Sánchez et al., 2014).

This is especially true considering the role of the highly dynamic and dramatic Pleistocene climatic cycles, which acted as a backdrop to human evolution in this period and have often been viewed as promoting population fragmentation and isolation, and thus leading to differentiation. These cyclical global environmental changes would have affected not only local conditions and survivability at any given point in time and space, but also enabled or hindered population movements through the availability of land bridges or ‘green corridors’ (e.g., ‘Green Sahara’; Larrasoaña et al., 2013, 2021) alternating with barriers (high sea levels, desert conditions) through time. These environmental processes would have resulted not only in repeated episodes of isolation, population fragmentation and adaptation to local conditions, accelerating differentiation, but also in population expansions and retreats leading to contact between formerly isolated groups, as well as extinctions and localized survival in refugia areas, peripatric and parapatric isolation and sympatric co-existence of distinct lineages. Such complex scenarios of repeated cycles of range expansion, contraction, and contact following environmental change are likely applicable to human evolution across Africa and Eurasia in the Middle to Late Pleistocene, resulting in the convoluted picture presented to us by the fossil record.

## 4 Open questions, ways forward

### 4.1 Modern human origins and diagnosing *H. sapiens* in the fossil record

Following these developments, current understanding of the origin of modern humans has shifted from previous models based on regional continuity (‘Multiregional Hypothesis’; e.g., Wolpoff et al., 1994) or replacement (strict ‘Out of Africa’ model; Stringer and Andrews, 1988). While the principal ancestry of modern humans is recognized to lie in Africa based on both genetic and fossil evidence, a small component of the genetic ancestry of all non-Africans is now understood to have originated from Neanderthals (ca. 2–3%; Prüfer et al., 2021) and, in some populations, also from Denisovans, who are discussed further below (up to 4% in Australian and New Guinean groups and up to 6% in some indigenous populations from the Philippines; Reich et al., 2010; Larena et al., 2021). Therefore models closer to the ‘replacement with hybridization’ (Bräuer, 1984) or ‘assimilation’ (Smith et al., 2005, 2017) scenarios are better suited to current knowledge (e.g., Aiello, 1993; Stringer, 2014). The timing of modern human origins has also been pushed back from between 200–100 ka to >300 ka, based on fossil (see below) but also genetic data, with deep modern human lineages coalescing around this time (e.g., Schlebusch et al., 2017; Bergström et al., 2020; Lipson et al., 2020). Original hypotheses of a modern human ‘birthplace’ in either Eastern or Southern Africa have given way to a more nuanced understanding of complex ancestry, possibly involving several distinct, geographically separated populations, which nevertheless interbred consistently through time (e.g., Gunz et al., 2009; Scerri et al., 2018; Klein, 2019). Finally, the evidence for early dispersals of early *H. sapiens* out of Africa has grown, and possible such events are now documented not only in the well-known sites of Qafzeh and Skhūl in modern-day Israel (ca. 130–90 ka; Bar-Yosef, 1998), but even earlier in Misliya, Israel (ca. 180 ka; Hershkovitz et al., 2018) and Apidima, Greece (>210 ka; Harvati et al., 2019).

Diagnosing ancestors of modern humans in the fossil record is not always straightforward. While the Neanderthal lineage has been characterized by an extensive list of derived features, or combinations of features, on which their diagnosis can be based (see review in Harvati, 2015), early (e.g., Day and Stringer, 1982, 1991) and more recent (e.g., Tattersall and Schwartz, 2008) attempts of developing diagnostic criteria for *H. sapiens* in the fossil record were shown to be too restrictive when compared to geographic or temporal variation of recent humans (see discussions in Pearson, 2008; Stringer, 2016). Nevertheless, a commonly cited set of *H. sapiens* traits based on the morphology of all living people includes increased skeletal gracility, an orthognathic face with reduced brow ridge and dentition, the presence of a bony chin throughout ontogeny, a large and globular neurocranium with elevated basicranial flexion, as well as aspects of dental microstructure, pelvic shape and, possibly, inner ear morphology (e.g., Stringer and Andrews, 1988; Stringer and Buck, 2014; Stringer, 2016; Beaudet et al., 2022). As with the Neanderthal lineage, these typical traits do not all occur at the same time in the fossil record, making the attribution of specific fossils to our own lineage increasingly difficult with increasing time depth (Stringer, 2016; Bergström et al., 2021). This results in a taxonomic dilemma: should the earlier segment of the *H. sapiens* lineage be recognized as a separate species (chronospecies), given that they do not possess the full anatomical configuration of the later representatives of their lineage (and if so, which one)? Or should they be referred to informally as, e.g., ‘early’ or ‘basal’ *H. sapiens* (Stringer, 2016; Stringer and Crété, 2022)?

The earliest currently recognized members of our lineage have been identified in Jebel Irhoud, Morocco, recently dated to ca. 300 ka (Hublin et al., 2017; Richter et al., 2017). Other potential early modern humans from Africa include the Florisbad specimen from southern Africa (Dreyer, 1935) and the Guomde specimen from eastern Africa, both dated to >180 ka (Bräuer et al., 1997); the Omo Kibish specimens (although Omo 2 exhibits more archaic morphology than Omo 1 and is sometimes considered to be distinct, possibly belonging to a more basal population of the *sapiens* lineage; see e.g., Day and Stringer, 1982, 1991; Klein, 2019; Sahle et al., 2019), recently redated to a context >230 ka (Vidal et al., 2022); and the Herto material from Ethiopia, dated to ca. 160 ka (White et al., 2003). Outside Africa potential representatives include the Western Eurasian/Mediterranean specimens Apidima 1, Greece, dated to >210 ka (Harvati et al., 2019); the Misliya 1, Qafzeh and Skhūl specimens, Israel, dated to ca. 180 ka (Hershkovitz et al., 2018) and between 130 and 90 ka (Grün, 2006), respectively; and several specimens from the Chinese cave sites of Zhiren, Fuyan, Huanglong, and Luna, dating between >100 and 70 ka (Liu et al., 2010a, 2010b, 2015; Shen et al., 2013; Bae et al., 2014). Not all these specimens exhibit the full suite of morphological features typical of modern humans, especially further back in time (and many are incomplete or even isolated elements). For example, the Irhoud specimens are characterized by modern humanlike facial, mandibular and dental anatomy, as well as aspects of their ontogeny (Smith et al., 2007; Hublin et al., 2017), but do not exhibit the globular neurocranium typical of modern humans, which appears among later specimens in mosaic fashion (e.g., Omo 1 vs. Omo 2; e.g., Harvati et al., 2019; Bergström et al., 2021).

Further complicating matters is the poorly understood polarity of some of these features, which has important implications for the taxonomy of individual fossils as well as the phylogenetic position of taxa. A case in point is modern human facial morphology, often considered derived for our lineage, but more recently interpreted as an ancestral retention (e.g., Lacruz et al., 2019). Such an interpretation would exclude specimens like Broken Hill 1 (recently dated to ca. 300 ka) and, consequently, *H. rhodesiensis*, from modern human ancestry as too derived in this respect (Grün et al., 2020). Alternatively, the Broken Hill specimen may represent a late survival of an ancestral taxon in eastern Africa, existing contemporaneously and perhaps also sympatrically with the early representatives of *H. sapiens*, in a similar fashion as the possible sympatry of *H. naledi* and early *H. sapiens* in southern Africa. Instances of interbreeding of modern humans with archaic lineages have now been inferred genetically also in Africa (Durvasula and Sankararaman, 2020; Lipson et al., 2020), a finding consistent with multiple lineages surviving alongside each other even after the differentiation of modern humans.

A better understanding of the early origins of modern humans will require overcoming major challenges, not least of which is the sparse African fossil record from this period and the lack of proper chronological placement of key fossils, as well as the lack of paleogenetic data, which could help resolve some of these questions. Given that most of the vast territory of Africa remains unexplored, systematic, targeted fieldwork aiming to recover stratified and potentially datable remains from currently little-explored regions should be a very high priority going forward. In addition, the development of molecular approaches that can be applied in lower latitude contexts will be crucial in addressing these questions and help remedy the existing high latitude bias in paleogenetic approaches.

### 4.2 Who were the Denisovans?

Denisova Cave in the Altai region of Russia offered unprecedented insight into hominin diversity when initial sequencing of endogenous DNA from a fifth distal phalanx revealed a mitochondrial sequence deeply divergent from Neanderthals and modern humans (Krause et al., 2010), later confirmed with mtDNA, as well as nuclear DNA, from additional specimens (Reich et al., 2010; Meyer et al., 2012; Sawyer et al., 2015). More recently, DNA recovered from sediment samples contributed to this knowledge (Slon et al., 2017). This lineage has been designated as ‘Denisovans’ without formal attribution to taxon (but see Derevianko, 2011 for proposal of *Homo altaiensis* nomen; also *Homo denisovensis* by Anagnostou et al., 2015; and *Homo denisensis* by Agadjanian and Shunkov, 2018).

A taxonomic attribution of the Denisovans is particularly intractable, given that most specimens assigned to this group are isolated teeth or undiagnostic bone fragments, and limited morphological information is currently available. This is a major obstacle in integrating this genetic lineage into the understanding of the region's complex known fossil record, for which limited molecular information is available. Indeed, the Asian human fossil record was poorly understood even before the advent of the Denisovans, comprising material attributed to *H. erectus* from both China and Indonesia, as well as specimens with proposed affinities to *H. heidelbergensis* s.l., in many cases characterized by poor chronological resolution and lack of context. As already mentioned, recent additions to the taxonomic diversity are the two recognized island species *Homo floresiensis* and *Homo luzonensis* (Brown et al., 2004; Détroit et al., 2019), while the presence of early *H. sapiens*, mostly represented by dental remains dating between ca. >100 and 70 ka, has also been proposed from several sites, mainly in southern China (Zhiren Cave: Liu et al., 2010a; Huanglong Cave: Liu et al., 2010b; Shen et al., 2013; Luna Cave: Bae et al., 2014; Fuyan Cave: Liu et al., 2015; see review in Reyes-Centeno, 2016). The addition of a genetic lineage, represented by minimal morphology, to the mix has further complicated matters, raising the question of which of the already known specimens/taxa might represent the Denisovan lineage.

What do we know so far about this group? Genetically, they share a more recent ancestor with Neanderthals than either of them do with modern humans (Meyer et al., 2012). Viable admixture with both Neanderthals and modern humans is evident by the genomic finding of a first-generation Denisovan-Neanderthal hybrid at Denisova Cave, identified from a nondiagnostic fossil fragment (Slon et al., 2018), and by the presence of Denisovan DNA in extant populations (Larena et al., 2021), respectively. The Denisovan geographic range is thought to have extended from the Altai region of Siberia all the way to Island Southeast Asia, a vast territory spanning multiple ecological zones. The southern extent of this range is inferred from the observation that some living populations in the Philippines, Melanesia, and Australasia share a relatively high proportion of Denisovan DNA in their genomes (Larena et al., 2021). Denisovan-like mitochondrial DNA was also found to characterize the Middle Pleistocene Sima de los Huesos specimens from Atapuerca, Spain (Meyer et al., 2014), which are otherwise similar to Neanderthals in their nuclear DNA (Meyer et al., 2016) and aspects of their morphology (e.g., Martinón-Torres et al., 2012). The Sima population is often considered ancestral to, or even early, Neanderthals (Arsuaga et al., 2014). This genetic similarity highlights the close relatedness of Denisovans to the early part of the Neanderthal lineage and *H. heidelbergensis* s.s., suggesting that they might also share morphological affinities.

The best potential insight into Denisovan anatomy thus far has been provided from a hemimandible fragment from the Tibetan Plateau site of Baishiya Karst Cave in Xiahe, China, dating to ca. 160 ka (Chen et al., 2019). The Xiahe mandible was attributed to Denisovans based on its proteome and constitutes the first evidence of Middle Pleistocene hominin presence in the high-altitude plateau, until recently thought to have been reached only by modern humans as late as ca. 40–30 ka (Chen et al., 2019). This attribution is supported by the later recovery of Denisovan-like sediment DNA from several layers of the same cave, albeit representing later periods, pointing to a long-term presence of this population (Zhang et al., 2020). The Xiahe jaw's morphology is consistent with some anatomical predictions made from comparative methylation maps of Denisovan, Neanderthal, and modern human genomes (Gokhman et al., 2019). Such genomic inference has further motivated the association of taxonomically contentious fossils like those from Xuchang or Xujiayao to Denisovans (Wu et al., 2022), while similarity with the dental morphology of the Xiahe mandible has led to the allocation of the lower molar from Tam Ngu Hao 2, Laos, to this lineage (Demeter et al., 2022)—although it is also compatible with a Neanderthal attribution.

The identification of Denisovan DNA in modern populations of island Southeast Asia raises the possibility that the fossil record of that region could potentially also represent Denisovan groups. Thus far, however, fossil-rich Middle Pleistocene sites are limited to Indonesia and are assigned to the *H. erectus* hypodigm, with the exception of the assemblage from Liang Bua, Flores, attributed to *H. floresiensis* (Brown et al., 2004) and the possibly related, much older fragmentary remains from Mata Menge (van den Bergh et al., 2016), as well as those from Callao Cave, the Philippines, attributed to *H. luzonensis* (Détroit et al., 2019). Together with the mandible fragment from the Penghu Channel of Taiwan (Chang et al., 2015), the Laos specimen is therefore the best candidate for Denisovans in Southeast Asia thus far. The expansive proposed Denisovan geographic and ecological range from Siberia to Southeast Asia, as well as their documented long-term presence on the Tibetan plateau and possession of high-altitude genetic adaptations, point to an impressive ecological tolerance and flexibility, a characteristic previously considered unique to our own taxon.

Beyond attempting to clarify a fascinating enigma, the ‘hunt’ for the Denisovans has brought the complexity of the Asian record into sharp focus. Matching specimens with preserved morphology to molecular evidence for the Denisovan lineage and extrapolating from there is a necessary first step to resolving not only the Denisovan question but also the broader issue of the Middle to Late Pleistocene human evolution in Asia. Improved chronological resolution of the existing record and new finds from controlled excavated contexts are also necessary. In our view, however, ultimately it will be the comprehensive and systematic analysis of the entire record, rather than individual fossils, which will significantly move the needle of our understanding of this complex picture.

### 4.3 Who is the common ancestor?

Current genomic estimates put the divergence of the last common ancestor of the modern human, Neanderthal, and Denisovan lineages at ca. 700–500 ka (see, e.g., Bergström et al., 2020). The fossil record during this period is relatively slim. Perhaps the most cited option for the last common ancestor of Neanderthals and modern humans (and therefore also Denisovans) is *H. heidelbergensis* s.l. The arguments put forth to support this idea include the striking morphological similarity between the African and European members of this taxon (Stringer, 1974, 1983; Harvati, 2007, 2009; Rightmire, 2008; Harvati et al., 2010), often interpreted as reflecting taxonomic uniformity; their broad chronological placement in the Middle Pleistocene; and their morphology, which has been interpreted as foreshadowing Neanderthal and modern human features in Europe and Africa, respectively (see, e.g., Rightmire, 2008). However, the discovery of earlier specimens in Iberia dating to ca. 850 ka, attributed to a different taxon, *Homo antecessor*, and exhibiting facial morphology until then considered derived for modern humans (Bermúdez de Castro et al., 1997; Freidline et al., 2013), has led to the re-examination of the polarity of these features and the resulting implications for the phylogenetic position of the African branch of *H. heidelbergensis* s.l.—whether recognized at a specific level (e.g., *H. rhodesiensis*) or not. This discovery prompted the proposal that *H. antecessor* may instead represent the last common ancestor (Bermúdez de Castro et al., 1997). In these reassessments, the facial morphology of specimens such as Bodo or Broken Hill (Kabwe) may be considered too derived to be ancestral to *H. sapiens* (e.g., Stringer, 2016; Bergström et al., 2021).

Efforts to approach these questions through evolutionary modeling have given mixed and even conflicting results. For example, Mounier and Mirazón Lahr (2016) supported a phylogenetic model of an early Middle Pleistocene (ca. 700 ka) common ancestor spanning both Africa and Europe and where the cranial shape of hypothetical virtual ancestors is similar to *H. heidelbergensis* s.l. (sensu e.g., Rightmire, 2008). This result is consistent with genetic estimates of divergence time, but implies higher rates of evolutionary change in the lineage leading to *H. sapiens*. Mounier and Mirazón Lahr (2016), however, also found inconsistencies between the models derived from facial vs. neurocranial data, with aspects of facial morphology pointing to exclusive affinities between Neanderthals and Middle Pleistocene specimens (as also postulated from the interpretation of facial features above). The authors proposed extensive evolutionary convergence in facial morphology as a possible solution because facial morphology has been proposed to be more labile than that of other cranial regions (e.g., Harvati and Weaver, 2006a). In another analysis of dental morphology using evolutionary rates and phylogenetic Bayesian approaches, Gómez-Robles (2019) rejected a Middle Pleistocene common ancestor, supporting a pre-800 ka divergence of the *H. sapiens* and *H. neanderthalensis* lineages instead. This work found that the highly derived Neanderthal-like dental morphology observed in Sima de los Huesos is incompatible with a Middle Pleistocene divergence of the Neanderthal and modern human lineages, unless exceptionally high dental evolutionary rates can be expected for this group. Such high rates could be explained by founder effect and isolation of this population, which could have accelerated the evolution of aspects of their morphology (Gómez-Robles, 2019), a scenario which may not have been uncommon in the Pleistocene (see above). These mixed results are further complicated by the inference of introgression between lineages (from modern human ancestors into the Neanderthal lineage) before 200 ka, as inferred from paleogenetic data (e.g., Posth et al., 2017; Chen et al., 2020; Peyrégne et al., 2022), which may also affect the expression of particular phenotypic traits, and which was not considered in the above analyses. It has been suggested, for example, that the phenotypic affinities between Neanderthals and Jebel Irhoud might signal a late Middle Pleistocene admixture event between lineages (Mounier and Mirazón Lahr, 2019).

In light of these developments, some authors have suggested looking deeper in the fossil record to find the last common ancestor. *H. antecessor* from Atapuerca, Spain, is one possibility. Other material which could belong to that taxon include the late Early to early Middle Pleistocene Tighenif specimens from Northern Africa or the even older Buia fossil from Eastern Africa (e.g., Stringer, 2016; Bergström et al., 2021). In our view, it is too early to exclude (at least the early part of) the Middle Pleistocene *H. heidelbergensis* s.l. from this role—or its African branch *H. rhodesiensis* from modern human ancestry. An assessment of variation in this record mainly with regard to possible sexual dimorphism (see, e.g., the more gracile face of Ndutu—a possible very early *H. sapiens* or, alternatively, female *H. heidelbergensis* s.l.—vs. the more robust Bodo or Broken Hill specimens; Stringer, 2016); an evaluation of the possibly differing rates of evolutionary change by anatomical region/feature (e.g., Harvati and Weaver, 2006a, Harvati and Weaver, 2006b; Reyes-Centeno et al., 2016, 2017); and a better understanding of the phenotypic impact of hybridization (see below) are all necessary to further evaluate and resolve phylogenetic relationships.

### 4.4 What is the impact of hybridization on the interpretation of the fossil record?

As paleogenetic analyses repeatedly point to hybridization and introgression as a relatively low level but potentially significant aspect of human evolution, it is imperative to understand the impact that these processes might have on the expression of phenotypic traits relevant to the interpretation of the fossil record. For example, understanding the polarity of morphological traits under the assumption of interbreeding between lineages would necessitate a more nuanced approach than in a traditional cladistic framework. This is because traits shared by two lineages, which normally would be interpreted as cases of primitive retention or convergence, might instead be explained as resulting from introgression. How to distinguish between these different options, however, is not straightforward, since there is no commonly accepted way of recognizing hybrids in the fossil record, the vast majority of which would not be first- or early-generation hybrids, but instead would represent admixed individuals with archaic genetic components inherited from interbreeding events many generations earlier.

Neanderthal-modern human hybrids proposed in the past based on skeletal morphology have faced criticism, with some cases provoking intense debate and even personal confrontation (e.g., Duarte et al., 1999; Tattersall and Schwartz, 1999a, Tattersall and Schwartz, 1999b). The most important shortcoming of such proposals has been the lack of understanding of how admixture might manifest in the phenotype, resulting in the absence of clear, testable criteria to recognize such cases (Harvati et al., 2007)—especially when interbreeding is proposed to have occurred many generations and thousands of years earlier (Harvati and Ackermann, 2022). An extensive list of studies of hybrid morphology among primates, both at the specific and subspecific levels, provide some guiding principles for the possible recognition of such cases (e.g., Cheverud et al., 1993; Schillaci et al., 2005; Ackermann et al., 2006, 2014, 2019; Ackermann, 2010). Effects of hybridization on the phenotype that might be investigated in the fossil record can include size differences (heterosis or dysgenesis) and transgressive shape compared to the parental taxa; mixed or intermediate morphologies; and developmental anomalies (see, e.g., Harvati and Ackermann, 2022). On a population level, groups including hybrid individuals are also expected to show increased variability, resulting from one or more of the above phenotypic effects on the individual level (Harvati and Ackermann, 2022). These effects, however, are more difficult to detect in later-generation hybrids, which are expected to be represented at higher frequencies than early-generation hybrids in the fossil record.

Recent work has aimed to investigate the morphology of known admixed individuals (from paleogenetic data) to better assess the likelihood of identifying signatures of interbreeding between lineages in the fossil record. These studies suggest that intermediate morphology, commonly proposed to indicate hybridization, is not necessarily associated with admixture, and that the effects of hybridization on the cranial phenotype may manifest differentially by cranial region. In their assessment of the 3D shape of the Oase 1 mandible, currently the fossil *H. sapiens* individual with the highest component of Neanderthal ancestry identified (Fu et al., 2015), Harvati and Roksandic (2016) found that only its large size and megadontia might flag this individual as potentially admixed, as its overall shape did not appear intermediate between modern human and Neanderthal morphologies. More recently, Harvati and Ackermann (2022) compared Eurasian Upper Paleolithic *H. sapiens* samples to Neanderthals and recent and ancient sub-Saharan Africans, chosen to represent the two parental populations, to investigate whether specimens with known Neanderthal ancestry components also manifest intermediate shape or developmental anomalies. This work highlighted the difficulties of deciphering the different evolutionary processes at work, as similar morphologies might result from different processes, including admixture, primitive retention, or convergence. No clear correspondence was found between the amount of Neanderthal genetic component and a Neanderthal-like shape on an individual basis (Harvati and Ackermann, 2022). Indeed, although Neanderthal genetic heritage was found to result in relatively more Neanderthal-like, elongated endocranial shapes among recent Europeans by Gunz et al. (2019), clearly demonstrating the potential impact of admixture on phenotypes even many generations later, this effect was linked not to overall amount of Neanderthal ancestry, but to the presence of specific alleles relevant for the expression of that morphology (Gunz et al., 2019). Harvati and Ackermann (2022) also found that different cranial regions seem to preserve phenotypic signals of admixture differentially, underlining the need to consider the evolutionary processes and constraints affecting different skeletal regions. They concluded that the consideration of multiple criteria such as intermediate and transgressive shape, transgressive size, and developmental anomalies can provide important hints as to the potential admixed status of individual fossils; but that these must be evaluated on a case-by-case basis, ruling out alternative scenarios like ancestral retentions or functional convergence when possible; and ideally verified with molecular data.

## 5 Conclusions

While the issues presented and discussed here do not represent the full extent of questions related to human evolution in the Middle to Late Pleistocene, this brief review has shown that, although the human fossil record of Middle Pleistocene *Homo* is significantly more abundant than it was half a century ago and innovative methodologies have greatly expanded our ability to study it, the greatest gains in our understanding are likely to result from a theoretical and conceptual shift toward more complex and nuanced evolutionary concepts for both species and speciation. Additional challenges include a better understanding of the chronological and paleoenvironmental framework of the Middle to Late Pleistocene, as well as the lack of integrative, synthetic study of the fossil record in its entirety. We expect new fieldwork and fossil discoveries to continue to bring novel insights, and for genomic and paleoproteomic approaches to play a central role in deciphering the record, especially if they can be developed to be applicable in lower latitude and earlier contexts. Gene annotation methods that link genotypes and phenotypes and their modularity in diverse environmental contexts (Gokhman et al., 2017; Brand et al., 2022), as well as evolutionary modeling approaches, will be particularly useful in the absence of a more complete fossil record. However, the envisioned integrative approach will only be possible through broad sharing of fossil data, something which is still rare in paleoanthropology, even after the development and widespread use of scan data, which can be shared digitally much more efficiently among researchers, more than two decades ago (Weber, 2001; Gibbons, 2002; Tattersall and Schwartz, 2002).

In closing, we emphasize that the bewildering, complex picture presented by human evolution in the Middle to Late Pleistocene should be seen as a feature, not a bug, reflecting evolutionary processes in all their messy glory. We look forward to the next unexpected discovery!

## Conflict of interest

The authors have no conflict of interest.
